# Erratum for “Incidence of acute kidney injury in dogs with systemic mycotic infections treated with amphotericin B (1996‐2020)”

**DOI:** 10.1111/jvim.16817

**Published:** 2023-07-25

Chan JC, Dear J, Palm C, Reagan K. Incidence of acute kidney injury in dogs with systemic mycoticinfections treated with amphotericin B (1996‐2020). J VetIntern Med. 2023;37(3):1030‐1037. doi:10.1111/jvim.16728

In the article cited above, the middle initial was omitted for the second author. The correct author names are shown below.

Jennifer C. Chan, Jonathan D. Dear, Carrie Palm, Krystle Reagan

We regret this error.

